# Induced pluripotent stem cell-derived lung alveolar epithelial type II cells reduce damage in bleomycin-induced lung fibrosis

**DOI:** 10.1186/s13287-020-01726-3

**Published:** 2020-06-03

**Authors:** Belén Alvarez-Palomo, Luis Ignacio Sanchez-Lopez, Yuben Moodley, Michael J. Edel, Anna Serrano-Mollar

**Affiliations:** 1 Banc de Sang i Teixits, Edifici Dr. Frederic Duran i Jordà, Passeig Taulat 116, 08005 Barcelona, Spain; 2 Department of Experimental Pathology, Institut d’Investigacions Biomèdiques de Barcelona, Consejo Superior de Investigaciones Científicas (IIBB-CSIC), Institut d’Investigacions Biomédiques August Pi i Sunyer (IDIBAPS), Barcelona, Spain; 3 Harry Perkins Research Institute, Centre for Cell Therapy and Regenerative Medicine (CCTRM), University of Western Australia, Perth, WA Australia; 4 Centro de Oftalmología Barraquer, Institut Universitari Barraquer, Universitat Autònoma de Barcelona, Barcelona, Spain; 5 Victor Chang Cardiac Research Institute, Sydney, NSW Australia; 6 Centro de Investigaciones Biomédicas en Red de Enfermedades Respiratorias (CIBERES), Madrid, Spain

**Keywords:** Induced pluripotent stem cells, Alveolar type II cells, Cell differentiation, Idiopathic pulmonary fibrosis, Cell therapy

## Abstract

**Background:**

Idiopathic pulmonary fibrosis is a chronic, progressive, and severe disease with a limited response to currently available therapies. Epithelial cell injury and failure of appropriate healing or regeneration are central to the pathogenesis of idiopathic pulmonary fibrosis. The purpose of this study is to investigate whether intratracheal transplantation of alveolar type II-like cells differentiated from induced pluripotent stem cells can stop and reverse the fibrotic process in an experimental model of bleomycin-induced lung fibrosis in rats.

**Methods:**

Human induced pluripotent stem cells were differentiated to alveolar type II-like cells and characterized. Lung fibrosis was induced in rats by a single intratracheal instillation of bleomycin. Animals were transplanted with human induced pluripotent stem cells differentiated to alveolar type II-like cells at a dose of 3 × 10^6^ cells/animal 15 days after endotracheal bleomycin instillation when the animal lungs were already fibrotic. Animals were sacrificed 21 days after the induction of lung fibrosis. Lung fibrosis was assessed by hydroxiprolin content, histologic studies, and the expression of transforming growth factor-β and α-smooth muscle actin.

**Results:**

Cell transplantation of alveolar type II-like cells differentiated from induced pluripotent stem cells can significantly reduce pulmonary fibrosis and improve lung alveolar structure, once fibrosis has already formed. This is associated with the inhibition of transforming growth factor-β and α-smooth muscle actin in the damaged rat lung tissue.

**Conclusion:**

To our knowledge, this is the first data to demonstrate that at the fibrotic stage of the disease, intratracheal transplantation of human induced pluripotent differentiated to alveolar type II-like cells halts and reverses fibrosis.

## One sentence summary

Transplantation of human iPSC-derived AEC2 reduces lung fibrosis, associated with reduced expression of α-SMA and TGF-β in the experimental bleomycin model in rats.

## Introduction

Idiopathic pulmonary fibrosis (IPF) is a chronic, progressive, and severe disease of unknown cause with a limited response to currently available therapies [1–3]. The median survival time is 3 to 5 years from the time of diagnosis [1]. Most patients show a progressive decline in pulmonary function leading to respiratory failure and death. The poor prognosis, combined with the scarcity of treatment options, provides a strong rationale for development of novel therapeutic alternatives for this disease [1].

Alveolar epithelial cell injury and failure of appropriate healing or regeneration are central to the pathogenesis of IPF. Epithelial cell damage and death result in breaks in epithelial basement membranes of alveoli. Subsequent migration of fibroblasts and myofibroblasts into the alveolar space through these gaps leads to intra-alveolar fibrosis with an exaggerated accumulation of extracellular matrix (ECM) components. The resulting fibrosis disturbs the normal lung architecture leading to lung dysfunction and respiratory failure [4–6].

Within the structure of the alveolar epithelium are the alveolar type II (AEC2) and type I (AEC1). One of the main important functions of AEC2 is the production, secretion, and recycling of pulmonary surfactant proteins that decrease the surface tension of the alveoli providing efficient ventilation and alveolar stability as well as acting as a protective barrier against possible infections [7, 8]. Under normal conditions, AEC2 have the ability of self-renewal and are the progenitor cell type for AEC1, the cells responsible for gas exchange in the alveoli [9, 10]. However, during the course of IPF, AEC2 undergo cell death and are replaced by myofibroblasts that show a great proliferative activity. In this regard, the death of the AEC2 and activation of fibroblasts or myofibroblasts are central in the development of the pulmonary fibrosis [11].

The loss of AEC2 prevents renewal and repairing of the alveolar wall and induces the secretion of fibrogenic mediators such as transforming growth factor beta (TGF-β). TGF-β acts as a major pro-fibrotic cytokine, potently increasing fibroblast recruitment, proliferation, differentiation into myofibroblasts, and production of ECM [12]. Myofibroblasts compose the majority of cells in the fibroblastic foci and are characterized by the presence of α-smooth muscle actin (α-SMA). They play an important role in the pathogenesis of pulmonary fibrosis by contributing to increased ECM deposition and reduced contractibility of lung tissue during IPF. Myofibroblasts also secrete TGF-β that in turn induces AEC2 apoptosis reducing the capability of wound healing and increasing lung architecture damage [11].

Cell transplantation therapy to treat lung disease has been proposed before; the most widely tested cell types are bone marrow mesenchymal stromal cells [13], but also other cell types such as cord blood mesenchymal stem cells, human amnion epithelial cells (hAEC) [14], and AEC2 in both animal and human clinical studies [15–17]. AEC2 are responsible for repairing the damaged alveolar epithelium in the event of lung damage and consequently proposed as an optimal cell type for the treatment of IPF. In preclinical studies, the administration of AEC2 during the inflammatory phase or during the fibrotic phase resulted in significant reduction of fibrosis and the restoration of surfactant levels [15, 16]. Moreover, in a clinical study performed with IPF patients, the intratracheal administration of heterologous AEC2 was safe, well tolerated, with no relevant side effects, stabilized disease progression, and improved health-related quality of life throughout a 1-year clinical follow-up [17]. Therefore, results of transplanting AEC2 have been promising in halting pulmonary fibrosis progression both in animals and in humans. Additionally, other minor lung cell populations have also been described as potential stem cells or distal epithelium that are activated in particular situations such as acute injury and are yet to be tested [18–20].

Induced pluripotent stem cells (iPSC) have been proposed as an alternative source of cells for cell therapy to treat lung disease with the possibility of increased cell production for clinical applications [21]. Differentiation of mouse or human iPSC to definitive endoderm and then into distal AEC2 and proximal lung cells has been well described before [22–25].

The aim of our study was to test in a rat model of bleomycin (BLM)-induced lung fibrosis if transplantation of iPSC-like AEC2 could improve the fibrotic phenotype and reconstruction of alveolar structure. Differentiation of iPSC resulted in high efficiency of surfactant protein C (SP-C)-positive lung cells of approximately 90–95%. The results demonstrate that iPSC-AEC2 intratracheal transplantation after 15 days of BLM instillation, when fibrosis had been fully developed, led to decreased collagen deposition and a significant decrease in both TGF-β expression and α-SMA expression. In summary, our results show a reduction in the severity of pulmonary fibrosis with iPSC-like AEC2 intratracheal transplantation at the fibrotic stage of development of the disease.

## Materials and methods

### iPSC preparation and its differentiation to AEC2

#### Culture of induced pluripotent stem cells

Human iPSC were purchased from ThermoFisher and cultured on vitronectin (ThermoFisher)-coated 6 well plates in Essential 8™ Flex Medium (Gibco). Cells were adapted to single cell passaging for 4–5 passages before starting the differentiation protocol.

#### Differentiation from iPSC to AEC2

The differentiation protocol from iPSC to AEC2 was adapted from previously published protocols by the Kotton group [24]. Single cell passaging-adapted iPSC were plated on vitronectin (ThermoFisher)-coated plates at 400,000 cells/well in 12 MW in Essential 8™ Flex Medium with Rock inhibitor. Twenty-four hours later (day 0), definitive endoderm was induced using STEMdiff definitive endoderm kit (StemCells Technologies) for 72 h (day 3). For the induction of anterior foregut endoderm, the cells were replated on human placenta collagen IV (Sigma)-coated 6 MW at about 200,000 cells/cm^2^ in basal differentiation media (BDM) (DMEM:F12 (3:1), 1 x B27, 0.5 x N2 (all from ThermoFisher), ascorbic acid 25 μg/ml (Sigma)) plus SB431542 10 μM (Merk) and Dorsomorphin 2 μM (Sigma) from day 3 to day 6. Rock inhibitor was added at day 3 and removed at day 4. For lung progenitor induction, the cells were transferred to BDM plus fibroblast growth factor-10 (FGF10) 10 ng/ml, keratinocyte growth factor (KGF) 10 ng/ml, bone morphogenetic protein-4 (BMP4) 10 ng/ml (all from Peprotech), retinoic acid 50 μM (Sigma), and CHIR99021 3 μM (Merk) from day 6 to day 15. At day 8, the cells were split 1:5. For the last step of AEC2 induction, the cells were transferred to BDM plus KGF 10 ng/ml and CHIR99021 3 μM from day 15 to day 22.

#### RNA extraction and cDNA synthesis for in vitro work

For RNA extraction, during the differentiation of iPSC to AEC2, in each stage, cells were washed with PBS and dissociated by adding trypsine solution or TrpLE™ select (ThermoFisher Scientific) to the plate. Cells were collected in a 15-ml tube and diluted with more medium, and the suspension then was centrifuged 5 min at 800 rpm. The pellet obtained was used for RNA extraction. RNA from cells was extracted following the PureLink^R^ mini Kit (Ambion) protocol and was quantified measuring the absorbance at 260 nm at Nanodrop ND-100 Spectrophotometer (Thermo Scientific). A 1% agarose gel was made to confirm that there was no degradation of RNA. The synthesis of cDNA was done using a kit (Bioline) and XP Cycler. We synthesized 1 μg for sample iPSC, definitive endoderm, day 15 and day 22. However, for AEC2, we synthesized 360 μg.

#### Quantitative real-time PCR for in vitro work

RT-PCR was performed with SYBR Green: 7.5 μl SYBR Green, 0.75 μl of each primer at 10 μM, 2 μl 10× PCR buffer, and 500 ng of RNA in 8 μl of water. Duplicates for each sample were performed in 96-well plates and analyzed for all the markers (GAPDH, Oct4, NKX 2.1, FOXA2, FOXA1, FOXP2, Sox9, SP-C, surfactant protein A (SP-A), p63, GATA6, and Aquoporin5). For definitive endoderm, we analyzed the following markers: CXCR4, C-Kit, Sox17, MEF2C (mesoderm specific was used as a negative control), and GAPDH. The real-time PCR reaction was performed with the 7500 Real Time PCR System (10 min at 95 °C, 40 cycles 10 s at 95 °C, and finally 40 cycles 34 s at 60 °C). RT-PCR experiments were run at Genetic Forensics Laboratory (Hospital Clinic, UB).

#### Fluorescence activated cell-sorting (FACS)

For flow cytometry, the samples were centrifuged 5 min at 200*g*, and then resuspended in PBS 5% Donkey serum-0.5% Triton X-100 and incubated 15 min at room temperature. After that, the samples were centrifuged again and resuspended in PBS 5% Donkey serum-0.1% Triton X-100 with the primary antibody in the correspondent sample. We left the samples for 30 min at room temperature. Next, secondary antibody (1:500) was added and left for 30 min at room temperature. Samples were washed two times with PBS and analyzed by flow cytometry.

#### Immunofluorescence

For immunofluorescence, iPSC, iPSC-AEC2 (day 22), and endogenous human AEC2 were previously plated in coverslips. Cells were fixed with 4% paraformaldehyde for 20 min. For immunofluorescence staining, cells were washed with PBS and blocked and permeabilized with PBS 6% Donkey serum, 1% BSA, and 0.5% Triton during 30 min at room temperature. Primary antibody used was mouse monoclonal anti-NKX 2.1 (1:250), anti-SP-C (1:50), anti-MUC (1:100), and anti- Oct4 (1:60). Samples were left over night at 4 °C rocking. The next day, cells were washed three times with PBS. Secondary antibodies were both prepared at the same dilution 1:200 (anti-rabbit Alexa488 and anti-mouse Alexa568). Samples were left during 1 h at 4 °C in the dark. After 1 h, DAPI was added for nucleus staining and incubated 10 min at room temperature. Finally, the slides were mounted on fluoromont and observed by confocal microscopy.

#### Alkaline phosphatase

For the reaction of alkaline phosphatase, the kit alkaline phosphatase blue kit (Membrane Substrate Solution-ThermoFisher Scientific) was used. Firstly, the cells were washed with PBS and 0.5 ml of paraformaldehyde 4% was added for 90 s. Then, cells were washed again with PBS and a mix of 250 μl of component A and 250 μl of component B was added and incubated for 30 min at room temperature. Slides were observed under a transmission microscope. AEC2 should be reactive for alkaline phosphatase activity and appear blue.

### Animals

Sprague-Dawley rats, weighting 200–225 g at the beginning of the experiment, were used, in accordance with the European Community (Directive 86/609/EEC) and Spanish guidelines for experimental animals, and it was approved by the institutional committees of animal care and research of University of Barcelona*.*

#### Bleomycin-induced lung fibrosis

Lung fibrosis was induced by intratracheal instillation of a single dose of BLM (2.5 U/kg) (Sigma, USA) dissolved in 400 μl of sterile saline under isofluorane anesthesia [15]. Control animals received the same volume of saline. The animal body weights were recorded every 2 days during the course of the experiment.

#### iPSC-AEC2 transplantation

At day 21 of differentiation, cells were labeled by incubating them for 20 min in F12 medium with Vybrant™ DiO Cell-Labeling Solution (ThermoFisher). The next day, the cells were lifted with 0.05% trypsin and were washed twice with saline solution and resuspended at 4.5 × 10^6^ cells/ml in saline solution the final concentration for transplantation. After 15 days of intratracheal BLM, each animal received 3.0 × 10^6^ cells (suspended in 400 μl of sterile saline) by intratracheal administration under isofluorane anesthesia. Control animals received the same volume of saline solution. The animals were sacrificed 21 days after the induction of lung fibrosis [15]. To avoid immunological rejection, all the transplanted animals started, the same day of cell administration, an immunosuppressive treatment with cyclosporine (Novartis) (10 mg/kg, orally) daily until the day of the sacrifice.

#### Experimental groups

The animals were randomly distributed into four experimental groups (*n* = 6 in each group): (1) control—saline instillation; (2) control + iPSC-AEC2 transplantation (C + iPSC-AEC2)—saline instillation + iPSC-AEC2 transplantation; (3) BLM—BLM instillation; and (4) BLM + iPSC-AEC2—BLM instillation + iPSC-AEC2 transplantation.

#### iPSC-AEC2 engraftment

Engrafted cells were assessed in whole lung single cell preparations. Lung tissue samples were digested with trypsin (Sigma, USA), chopped into 1–2 mm^2^ cubes, treated with 75 U/ml DNase (Sigma, USA) dissolved in saline, and filtered through nylon meshes ranging from 150 to 30 μm. The resulting cell suspension was centrifuged 10 min at 500*g* and then washed twice with PBS and analyzed by AMNIS Image StreamX flow cytometry. Moreover, cell engraftment was also evaluated by fluorescent microscopy. Before cell transplantation, cells were labeled by the Vybrant™ DiO Cell-Labeling Solution (ThermoFisher) following the manufacturer’s protocol. At the end of the experiment, the lungs were collected, frozen, and embedded in OCT (Jung, Japan). The nuclei were stained with DAPI.

### Fibrosis measurement

#### Hidroxyproline content

Lung hydroxyproline content was measured as an indicator of collagen deposition, following the method outlined by Woessner [26]. Samples were homogenized and then hydrolyzed in 6 M HCl, and the hydrolysate was then neutralized with 2.5 M NaOH. Hydroxyproline in the hydrolysate was assessed colorimetrically at 550 nm with *p-*dimethylaminobenzaldehyde. The results are expressed as micrograms of hydroxyproline per lung.

#### RNA extraction and cDNA synthesis for in vivo work

To preserve the native ratio between DNA and RNA, Selfie-qPCR analysis was performed using sample lysate without nucleic acid extraction as previously described [27]. Briefly, tissue was homogenized in 100ST DNA/RNA/Protein Solubilization Reagent (#DCQ100ST, DireCtQuant) 5 μl/mg. The homogenized tissue was incubated at 90 °C for 3 min with 750 rpm agitation, centrifuged at 10,000*×g* for 10 min, and the supernatant was used directly to measure mtDNA, 7SDNA, nuclear DNA, and mtRNA as described [27].

#### Strand-specific transcription quantification by Selfie-qPCR

Strand-specific analysis of mtDNA transcription was performed by Selfie-qPCR as previously described, adapting the method to qPCR [27]. This method enables separate analysis of transcriptional activity of each one of the DNA strands without using a reference gene. The Selfie-qPCR procedure includes three steps: (1) sample and mtRNA strand-specific primer pre-annealing in duplicate aliquots of the same sample, (2) reverse transcription with retro-transcriptase enzyme in one duplicate and no enzyme in the other duplicate, and (3) qPCR analysis. To anneal the primers to their complementary transcripts, a reaction mixture containing the sample and 500 nM primer in 10 μl of double-distilled water was heated to 70 °C for 1 min, followed by a gradual decrease of temperature to 22 °C. Afterwards, we added 4 μl of reaction buffer 5× (EP0751, ThermoFisher), 2 μl 10 mM dNTPs (R0191, ThermoFisher), 0.5 μl Ribolock RNase inhibitor (EO0381, ThermoFisher), and double-distilled water to a final volume of 19.5 μl to each duplicate. After mixing both tubes well, we added 0.5 μl of Maxima H Minus reverse transcriptase (EP0751, ThermoFisher) to one of the duplicates and 0.5 μl of enzyme storage buffer to the second duplicate. Then, both tubes were incubated at 60 °C for 30 min to perform the retro-transcription, followed by 90 °C incubation for 3 min, to inactivate the reverse transcriptase. Next, 4 μl of each duplicate was added to a qPCR reaction mixture containing 100 nM of the corresponding primer, 1× EvaGreen ddPCR Supermix, in a final volume of 20 μl. qPCR was performed in a thermal cycler (C1000 Touch Thermal Cycler, Bio-Rad) using the following thermal profile: 95 °C 5 min, (95 °C 30 s; 60 °C 1 min) 40 repeats, 4 °C 5 min, and 90 °C 10 min. Non-template controls containing all the reagents and the corresponding amount of solubilization buffer without sample lysate were included in all steps of the procedure. The number of mtRNA transcripts was calculated by subtracting the amount of amplicons measured in the reaction without reverse transcriptase (RT−) from the reaction with reverse transcriptase (RT+) and dividing by (RT−). The used primers for TGF-β were forward 5′GACTCTCCACCTGCAAGACC3′ and reverse 5′GGACTGGCGAGCCTTAGTTT3′ and for α-SMA were forward and reverse forward 5′CATCACCAACTGGGACGACA3′ and reverse 5′TCCGTTAGCAAGGTCGGATG3′.

#### SDS-PAGE and Western blot

Protein samples were extracted using Nonidet P-40 buffer. SDS-PAGE was performed on 5–13% acrylamide gels. Proteins were electrotransferred to nitrocellulose membrane and probed with primary antibodies. The antibodies used included mouse anti-α-SMA (Acris Antibodies, Germany), molecular weight 42 kDa, and mouse anti-β-actin (Sigma, USA), molecular weight 42 kDa, which served as a housekeeping reference. The membranes were incubated with the corresponding peroxidase-conjugated secondary antibodies, washed, and then incubated with ECL reagents (GE Healthcare Europe GmbH; Freigburg; GE) before exposure to high-performance chemiluminescence films. Gels were calibrated using Bio-Rad standard proteins (Hercules, CA) with markers covering a 7–240-kDa range.

Films were scanned by using image-editing software NIH ImageJ software for densitometric analysis of immunoreactive bands.

#### Immunostaining studies

For the immunofluorescence studies, the lungs were collected, frozen, and embedded in OCT (Jung, Japan). Eight-micrometer tissue sections were obtained, and the α-SMA protein was assessed using an antibody against α-SMA (Abcam, UK) and revealed with a secondary antibody anti-mouse FITC (Acris, Germany). The α-SMA was observed in green color. The nuclei were stained with DAPI. Tiling fluorescent microscopy was used to assess more than 10% of each section and 2000 cells counted for each condition.

#### Histology

The lungs used for histology were inflated and fixed with 10% neutral buffered formalin, immersed in the fixative solution for 24 h, and paraffin-embedded. Four-micrometer sections were stained with hematoxylin-eosin and Masson’s trichrome to identify inflammatory cells, connective tissue, and collagen deposition.

### Statistical analysis

Data are expressed as mean ± SEM values with 95% confidence intervals (CIs). Statistical analysis was carried out by analysis of variance (ANOVA) followed by appropriate post hoc tests, including the Newman-Keuls test when differences were significant (GraphPad Software Inc., USA). A *p* value of < 0.05 was considered significant.

## Results

### Differentiation of human iPSC to AEC2

**Fig. 1:**
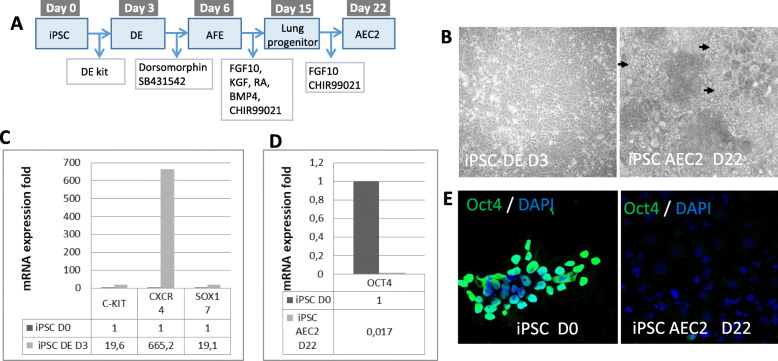
**a** Schedule of the differentiation protocol from iPSC to AEC2. **b** Open field photographs of the iPSC during differentiation, at DE stage (day 3) and AEC2 stage (day 22). The arrows indicate typical epithelial shape cells. **c** RT-PCR data for DE markers of cells at day 3. **d** RT-PCR data for pluripotency marker OCT4 of cells at day 22. **e** Immunofluorescence staining for OCT4 (green) in iPSC at day 0 and iPSC-AEC2 at day 22. Nuclei were stained with DAPI (blue). DE, definitive endoderm; AFE, anterior foregut endoderm; iPSC, induced pluripotent stem cells; AEC2, alveolar type II cells

For the production of iPSC-derived AEC2, we used a commercially available human iPSC line (cord blood cells reprogrammed with episomal vectors, ThermoFisher) and applied a previously published differentiation protocol to AEC2 described by the Kotton group [24] (Fig. 1a). Cells were adapted to single cell passaging and differentiated to definitive endoderm using a commercial kit (Stem Cell Technologies). After 3 days, the cells had acquired the characteristic polygonal shape of definitive endoderm and RT-PCR analysis showed enhanced expression of typical definitive endoderm markers: C-KIT, CXCR4, and SOX17 (Fig. 1c). Definitive endoderm cells were then replated and transferred sequentially to anterior foregut endoderm induction medium, lung progenitor induction medium, and finally AEC2 induction medium. At day 22, the end of the differentiation protocol, the cells had changed significantly in morphology and looked clearly epithelial (Fig. 1b). To check that no undifferentiated iPSC were left at day 22, we assessed the expression of the pluripotency marker Oct3/4 and found reduced gene expression (Fig. 1d) and protein levels in differentiated cells (Fig. 1e).

### Characterization of human iPSC-AEC2

**Fig. 2:**
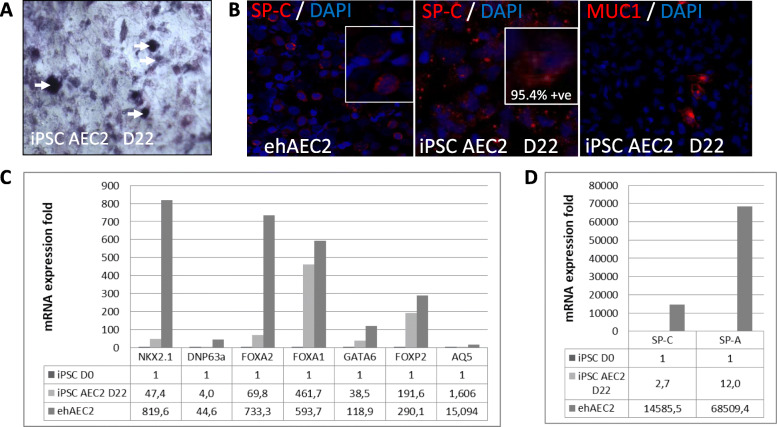
**a** Alkaline phosphatase staining (blue) of iPSC-AEC2 at day 22. The arrows indicate alkaline phosphatase-positive cells. **b** Immunofluorescence staining for SP-C (red) in ehAEC2 and in iPSC-AEC2 at day 22, and MUC1 (red) iPSC-AEC2 at day 22. Nuclei were stained with DAPI (blue). **c** RT-PCR data for AEC2 cell markers (NKX2.1, DNP63a, FOXA2, FOXA1, GATA6, FOXP2) and AEC1 cell markers (AQ5) of iPSC at day 0 and day 22 and of ehAEC2 cells. **d** RT-PCR data for AEC2 markers (SP-C, SP-A) of iPSC at day 0 and iPSC-AEC2 at day 22 and of ehAEC2 cells. iPSC-AEC2, induced pluripotent stem cells derived to alveolar type II cells; AEC2, alveolar type II cell; AEC1, alveolar type I cell; ehAEC2, endogenous human AEC2; SP-C, surfactant protein C; SP-A, surfactant protein A

To demonstrate that the differentiated cells had acquired an AEC2 phenotype, we first stained for alkaline phosphatase activity, a well-established general marker for AEC2, and observed clear foci of alkaline phosphatase-positive cells (Fig. 2a). Next, we analyzed the capacity of the cells to express typical AEC2 markers. At the protein level, we saw that iPSC-AEC2 at day 22 expressed SP-C in the cytosol and accumulated in granules, with resemblance to the pattern observed in endogenous human AEC2 from donor lung (Fig. 2b). We also stained for MUC1, an epithelial marker that is mostly found in airway cells, and observed sporadic expression indicating that the population was not totally pure alveolar (Fig. 2b). Next, we assessed gene expression levels of a battery of genes typically expressed in AEC2: NKX2.1, P63, FOXA2, FOXA1, GATA6, FOXP2, SP-C, SP-A, and AEC1 typical marker AQ5. All samples demonstrated increased gene expression (Fig. 2c). FOXA1 and FOXP2 gene expression levels were found to be comparable to those of endogenous human lung AEC2. Those marker indicating more mature alveolar cells, SP-C and SP-A, were overexpressed to modest levels compared to endogenous AEC2, indicating that iPSC-AEC2 were probably not fully mature.

### Transplantation of human iPSC-AEC2 into the lungs of bleomycin rat model

**Fig. 3:**
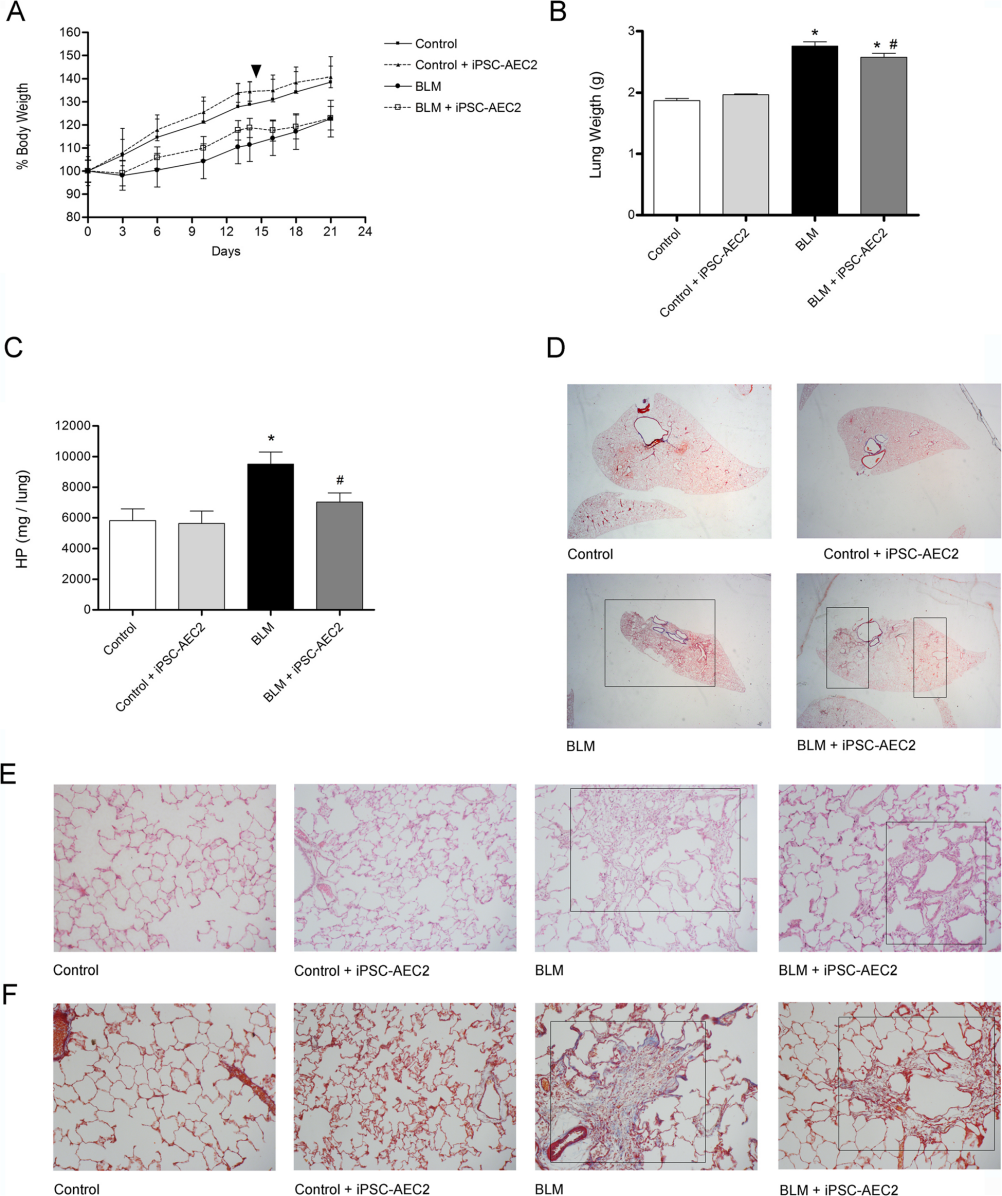
**a** Curves of animal body weight over time. On day 0, the animals received bleomycin (BLM). The arrowhead indicates the day of iPSC-AEC2 cell transplantation (day 14). Data are means ± SEM of 6 animals per group (*p < 0.05 vs control groups). **b** Lung weight at the end of the experiment (21 days). iPSC-AEC2 cell transplantation reduced significantly the lung weight compared to the BLM group. Data are means ± SEM of 6 animals per group (*p < 0.05 vs control groups, ^#^*p* < 0.05 vs BLM group). **c** Lung hydroxyproline (HP) levels at the end of the experiment (21 days). iPSC-AEC2 cell transplantation reduced significantly the total lung content of hydroxyproline compared with the BLM group. Data are means ± SEM of 6 animals per group (*p < 0.05 vs control groups, ^#^*p* < 0.05 vs BLM group). **d** Representative photographs of lung sections stained with Masson’s trichrome from all the experimental groups. Insets show changes in the multifocal parenchymal lesions in BLM non-transplanted lungs and BLM iPSC-AEC2 transplanted lungs. **e** Representative photomicrographs of lung sections stained with hematoxylin-eosin; iPSC-AEC2 transplantation in fibrotic animals was able to ameliorate the inflammatory and pulmonary lesions (inset). Magnification × 200. **f** Representative photomicrographs of lung sections stained with Masson’s trichrome; the presence of interstitial collagen (blue staining, inset) was also diminished in the BLM + iPSC-AEII group. Magnification × 200

**Fig. 4:**
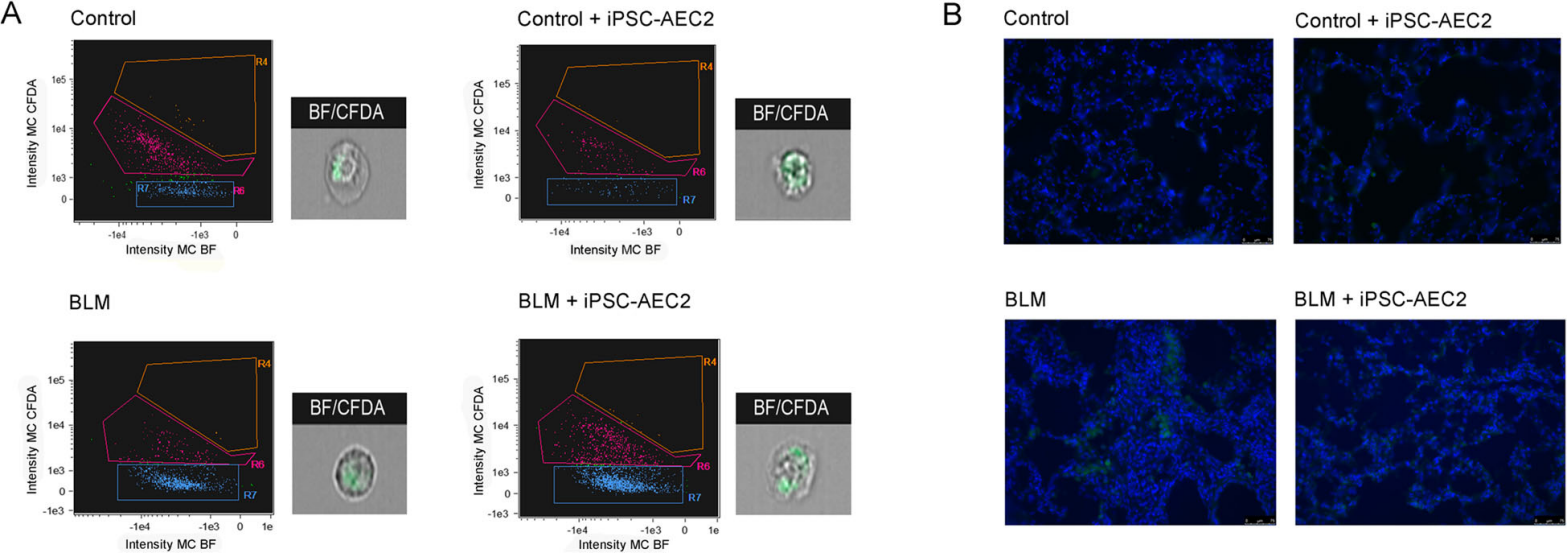
**a** Flow cytometry AMNIS Image StreamX analysis of whole lung single cell preparations. Histograms and photo of cells from flow cytometry by AMNIS Image StreamX analysis of whole lung single cell preparations to detect transplanted cells in rat lungs after 1 week transplantation. Control and control + iPSC-AEC2 groups where no labeled cells were BLM and BLM + iPSC2 groups demonstrating that no positive cells (zone R4) could be found 1 week after transplanting in the rat lung. MC_BF, Multi-Channel_Bright Field; MC_CFDA, Channel_Vybrant™ Cell Fluorescent Dye; MC, Multi-Channel. **b** Detection of iPSC-AEC2 engraftment after cell transplantation by fluorescence microscopy. For tracking cell purposes, iPSC-AEC2 cells were labeled with green with Vybrant™ DiO. Nuclei were stained in blue with DAPI. No green cells were observed in the transplanted groups over background/autofluorescence signal in the rat lungs (control + iPSC-AEC2 and BLM + iPSC-AEC2). Magnification × 400

BLM instillation of rat lungs initially causes a loss in body weight that normally recovers, and we observed that iPSC-AEC2 transplantation did not modify the body weight with time (Fig. 3a). However, the lung weight showed a significant decrease in the iPSC-AEC2 transplanted group compared with the BLM group, suggesting less fibrosis (Fig. 3b). Therefore, the amount of hydroxyproline, a modified amino acid specifically found in collagen, was assessed to determine how iPSC-AEC2 transplantation could alter the natural course of BLM-induced lung injury. BLM lungs showed a significant increase in the amount of lung hydroxyproline when compared with the control groups (Fig. 3c). In contrast, levels of hydroxyproline were significantly reduced in the BLM + iPSC-AEC2 transplanted group which did not significantly differ from levels observed in the control groups (Fig. 3c). These results confirm that transplantation of iPSC-AEC2 induces a reduction in collagen deposition and as a consequent of the fibrotic response. To further examine the effect of iPSC-AEC2 transplantation in BLM-induced pulmonary fibrosis, serial lung sections were stained with hematoxylin-eosin or Masson’s trichrome (Fig. 3d–f). To have a whole vision of lung tissue, Masson’s trichrome-stained lung sections were examined by stereomicroscopy that illustrates the heterogeneous topography of the fibrotic lesions (patchy areas of lung fibrosis) (Fig. 3d). BLM lungs showed extensive areas of fibrosis compared with BLM + iPSC-AEC2 transplanted lung that had less fibrosis (Fig. 3d). Moreover, fibrotic lesions were examined by light microscopy, and lung tissue sections from control rats showed no evidence of inflammation or epithelial damage. As expected, lung tissue sections from rats with BLM-induced fibrosis showed marked peribronchiolar and interstitial infiltration with inflammatory cells, extensive cellular thickening of interalveolar septa, interstitial edema, increased interstitial cells with a fibroblastic appearance, and excessive collagen deposition (Fig. 3e, f). Although multifocal parenchymal lesions and septal widening were still present in the lungs transplanted with iPSC-AEC2, the organized fibroblast foci were smaller and considerably less frequent than were observed in the BLM lungs. The reduction in parenchymal lesions is clearly observed with large areas of undamaged tissue with normal alveolar architecture (Fig. 3e, f). Compared with the BLM group, BLM + iPSC-AEC2 transplanted animals showed less edema and less collagen deposition. Assessment of cell engraftment of transplanted human iPSC-AEC2 in single cell preparations of the rat lung tissue by AMNIS Image StreamX flow cytometry and by fluorescent microscopy failed to detect any cell engraftment (Fig. 4), suggesting a paracrine effect.

### Transplanted human iPSC-AEC2 reduce α-SMA-positive cell fibrosis

**Fig. 5:**
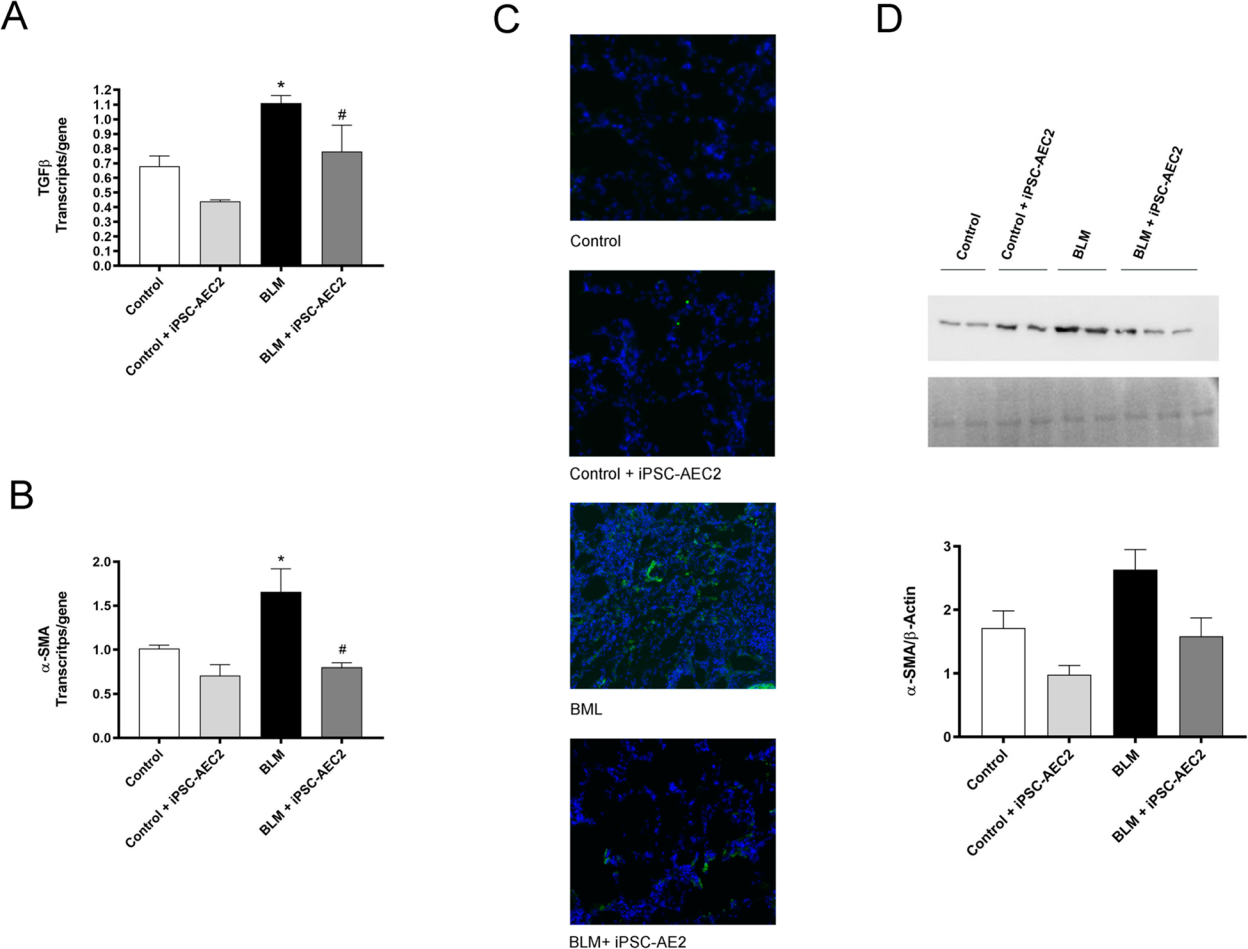
**a**, **b** Changes in the mRNA expression of markers for fibroblast activation (TGF-β and α-SMA) were evaluated by Selfie-qPCR. Data are means ± SEM of 6 animals per group (*p < 0.05 vs control groups, ^#^*p* < 0.05 vs BLM). **c** Immunohistological staining with anti-α-SMA (green) shows the presence of α-SMA-positive fibroblast in BLM animals. iPSC-AEC2 cell transplantation reduces the number of α-SMA-positive cells. (magnification × 400). **d** Graph of integrated optical density (area × density) of each band using Image-Pro plus software for α-SMA analyzed by immunoblot and normalized to β-actin (*n* = 2 for control, control + iPSC-AEC2, and BLM; *n* = 3 for iPSC-AEC2). Data are means + SEM (*n* = 2 for control, control + iPSC-AEC2, and BLM; *n* = 3 for iPSC-AEC2)

To confirm the decrease in fibrosis induced by iPSC-AEC2 transplantation, we studied the changes in gene expression of fibrotic markers TGF-β and α-SMA. Quantitative real-time PCR analysis revealed a significant increase in the expression of both markers in the BLM group, compared to the control group (Fig. 5a, b). iPSC-AEC2 transplantation in fibrotic animals significantly decreased gene expression levels of TGF-β and α-SMA compared to control levels (Fig. 5a, b). High levels of α-SMA in BLM animals indicate an increase in the amount of myofibroblasts that are directly related to fibrosis. In this regard, and in order to evaluate one of the mechanisms involved in the beneficial effect of iPSC-AEC2 transplantation, we also evaluate the presence of myofibroblasts and the α-SMA protein release. Results showed that iPSC-AEC2 transplantation strikingly reduced the number of α-SMA-positive cells and the levels of α-SMA release in the lung tissue of transplanted animals compared with the fibrotic BLM animals (Fig. 5c, d). The results indicate that one of the mechanisms involved of the positive effect of transplanted iPS-AEC2 is related with a significant reduction in α-SMA and TGF-β gene expression.

## Discussion

In the last decade, different cell therapies using various types of cells have been proposed as prospective novel IPF treatments. In this study, we evaluated the therapeutic effect of iPSC-AEC2 after 15 days of BLM administration in rats, when fibrosis had already developed in the animal lungs. We demonstrate that intratracheal transplantation of AEC2 derived from iPSC is able to decreased lung fibrosis. The discovery and development of new technologies to treat lung disease that includes IPF are essential because of a limited repertoire of currently available therapies [1–3].

The timing for cell transplantation is a critical factor determining the effect of therapy. The development of the BLM-induced pulmonary fibrosis model has three different well-recognized phases. In the first 3 days after BLM instillation, an acute inflammatory response is triggered; after 7 days, fibroblasts begin to proliferate; after 15 days, the disease is already fully developed [15]. This last phase is characterized by the loss of lung architecture due to remodeling process, where there is an increase in the deposition of ECM components together with a large proliferation of fibroblasts and α-SMA-positive cells [15]. Over the last decade, a great number of preclinical studies based in cell therapies have been conducted as prospective treatment for IPF. Various types of cells have been used, including different stem cells such as bone marrow-derived mesenchymal cells, hematopoietic stem cells, adipose stem cells, embryonic stem cells, iPSC, and lung stem cells [13, 28, 29]. Additionally, AEC2 and lung mixed epithelial cells have also been investigated [13, 28, 29]. Usually, most cell replacement therapy strategies have been assayed as a pre-treatment, after 24 h of BLM administration or during the early stages of fibrosis development 3 or 7 days after BLM [13, 28, 29]. In general, cell administration during these initial stages of the disease showed very promising results, because many of these cell types are able to inhibit the inflammatory response, leading to the slowdown of fibrogenesis. However, some stem cell therapies demonstrated controversial results related to efficacy when cells were administered during the fibrotic phase and even shown some aberrant action [28, 29]. This could be explained because stem cells could differentiate into fibroblasts and therefore most likely increase the development of fibrosis [28, 29]. Nevertheless, the administration of AEC2 at 3, 7, and 15 days after BLM administration demonstrated a reduction in fibrosis even in the fibrotic phase [15, 16]. The use of lung progenitor cells or differentiated lung cells would explain why this cell type increases the effectiveness during the fibrotic phase. Consistent with these latest studies, our results also demonstrated that iPSC-AEC2 transplantation is able to significantly reduce collagen deposition when fibrosis is already formed. Moreover, the transplanted iPSC-AEC2 inhibited the expression of TGF-β and α-SMA in the animal lung tissue. TGF-β is a powerful pro-fibrotic factor, and its overexpression induces ECM protein synthesis and regulates the differentiation from fibroblasts to myofibroblasts. At the same time, myofibroblast activation requires the presence of ECM proteins and TGF-β. The decreased expression of TGF-β and α-SMA observed after iPSC-AEC2 transplantation can in turn diminish fibrosis by decreasing the expression of collagen, myofibroblast differentiation, and proliferation. This is associated with a decrease in fibrotic lesions and lung alveolar architecture improvement. As we stated above, most cell therapies have been conducted during the early stages of fibrosis development, and therefore, it is difficult to compare our work with previous studies. To date, few of the reported studies have been performed with IPSC derived into lung lineage cells [30–32]. In those studies, iPSC-AEC2 were administered during the first 24 h or 3 days after BLM administration and both studies reported a decrease in inflammation and fibrosis [30–32]. Although these works agree with our results, it is important to take into account that they were not performed during the fibrotic phase of the disease development.

We exhaustively searched for evidence of transplanted iPSC-AEC2 at the time of sacrifice of the animals by single cell flow cytometry and by fluorescent microscopy of dissected lung tissue. The absence of the implanted cells 1 week after the transplantation suggests that the cells failed to engraft as a true cell replacement therapy, despite immune suppression treatment of animals. These results do not correspond as reported by other research conducted with iPSC-AEC2 or ACE2 where cell engraftment was observed; however, the percentage of engrafted cells in those studies was not stated [15, 16, 30–32]. Booh and colleagues reported only 1% of cells engraft after transplantation [31]. In our work, although engrafted cells were not found in the lungs after 1 week of transplantation, beneficial effects were observed that could be due to several aspects: (i) loss of cell dye, (ii) iPSC-AEC2 were replaced by endogenous progenitor cells, or (iii) the cells were not engrafted and the positive effects were due to a paracrine effect. In agreement with the latter scenario, it has been reported that the administration of conditioned medium from iPSC was able to reduce the levels of inflammatory cytokines, chemokines, and collagen deposition [33]. This warrants further investigation in our model whether there is a real engraftment of the cells or if there is a paracrine effect of the transplanted cells.

Another key challenge in the field of cell replacement therapy strategy that must be addressed is the possibility of chromosomal mutations that would lead to the formation of tumors post-cell transplantation into the lung [34]. In our study, we did not observe any tumors in the transplanted lungs, albeit at a short time point after cell transplantation. Accordingly, other studies have also not found evidence of tumors in the lung, even after 12 months of iPSC-AEC2 transplantation [31].

Although our protocol for cell differentiation from iPSC into AEC2 has been effective with high efficiency, it is important to develop further a protocol where the differentiation will be fully efficient. Our iPSC differentiated into AEC2 did not fully resemble endogenous lung AEC2 by expression of some specific gene markers, in particularly SP-C expression levels. Further development of the protocol and niche mimicking, which might only be achieved inside the lung architecture, is warranted. Reaching the full mature phenotype is hard to achieve because it may demand using lung niche mimicking strategies such as (i) architecture, (ii) a dynamic fluid flow, or (iii) transplantation in vivo. Possible modifications of the protocol to improve the maturation could potentially use 3D gels or exposing the AEC2 to an air-liquid interphase in the final part of the differentiation protocol. For example, the group of Kotton has designed 3D alveolospheres that are found to outperform 2D cultures in AEC2 differentiation and functionality [24]. However, we considered that for the purpose of transplantation, single cells would be better to reach the thin passages of alveoli. This warrants future work to define and using 3D lung structures in future studies to develop advanced iPSC-AEC2 stem cell replacement therapy to treat lung disease.

There are multiple therapeutic applications of this work. Our results indicate that iPSC-AEC2 is a promising new advanced cell therapy because the isolation of lung stem/progenitor AEC2 from the lung is a complicated process with limited availability. The use of iPSC differentiated to AEC2 is an easy and efficient approach for the lung cell-based therapy that allows for autologous cell transplantation. In addition, the derivation of iPSC bypasses the ethical concern associated with the use of human embryonic stem cells and provides an alternative source of cells that can be used as donor cells for cell therapy. Alternatively, iPSC banks from HLA homologous donors could provide HLA-matched lung cells that would involve minimal immune rejection of the grafted cells.

In conclusion, this a first study to transplant iPSC-AEC2 15 days after BLM instillation during the fibrotic stage of the disease. We confirm that human iPSC-derived AEC2 can acquire the AEC2 phenotype in vitro. Intratracheal transplantation of iPSC-AEC2 is able to reduce pulmonary fibrosis when fibrosis has already developed, decreasing the amount of collagen by the inhibition of both TGF-β and α-SMA expression.

## Data Availability

All the data supporting the findings will be made public and can be shared by contacting the corresponding authors ASM and MJE.

## Abbreviations

IPF: Idiopathic pulmonary fibrosis
ECM: Extracellular matrix
AEC2: Alveolar type II cells
AEC1: Alveolar type I cells
TGF-β: Transforming growth factor beta
α-SMA: α-Smooth muscle actin
hAEC: Human amnion epithelial cells
Krt5: Keratin 5
BLM: Bleomycin
iPSC: Induced pluripotent stem cells
SP-C: Surfactant protein C
SP-A: Surfactant protein A
MW: Multi-well cell culture plate
BDM: Basal differentiation media
FGF10: Fibroblast growth factor-10
KGF: Keratinocyte growth factor
BMP4: Bone morphogenetic protein-4
MC_BF: Multi-Channel_Bright Field
MC_CFDA: Channel_Vybrant™ Cell Fluorescent Dye
MC: Multi-Channel
